# Gender-Specific Risk Factors Contributing to Mortality in Patients Hospitalized With Alcoholic Cirrhosis

**DOI:** 10.7759/cureus.16271

**Published:** 2021-07-08

**Authors:** Youseung Kim, Shravani Reddy, Mohamad Mouchli, Robert Summey, Chirstopher Walsh, Adil Mir, Lindsey Bierle, Marrieth Rubio

**Affiliations:** 1 Internal Medicine, Virginia Tech Carilion School of Medicine, Roanoke, USA; 2 Gastroenterology and Hepatology, Virginia Tech Carilion School of Medicine, Roanoke, USA; 3 Gastroenterology and Hepatology, Cleveland Clinic Foundation, Cleveland , USA; 4 Medicine, University of Pennsylvania, Philadelphia, USA; 5 Internal Medicine, Carilion Clinic, Ronaoke , USA

**Keywords:** alcoholic cirrhosis, gender comparison, cirrhosis mortality, alcohol related cirrhosis, alcohol-related liver disease, gender disparities

## Abstract

Introduction

Identification of gender-specific prognostic factors in patients with alcoholic liver cirrhosis (ALC) is integral to understanding disease severity and mortality rates. We gathered data on various widely-used laboratory values and comorbid conditions among male and female patients with ALC after initial hospitalization. These individual risk factors were assessed for their relationship with mortality based on gender.

Methods

We performed a retrospective observational study of hospitalized patients with either a new or prior diagnosis of ALC from 2008 to 2016 with follow-up through June 2018. The electronic medical record (EMR) was queried for demographics, comorbidities, lab values, and mortality. The cumulative risks of mortality after the first hospitalization were estimated using Kaplan-Meier curves and compared among both genders. Demographic data, lab values, and comorbidities associated with cirrhosis were assessed using multivariate Cox proportional hazard analysis to determine risk factors associated with mortality.

Results

We identified 247 male patients (mean age 54.19 ± 13.14 years) and 78 female patients (mean age 51.10 ± 11.60 years) hospitalized at Carilion Clinic with a diagnosis of ALC. About 70% (male) and 46% (female) endorsed alcohol use at the time of admission, 10% (male) and 13% (female) endorsed illicit drug use, and 56% (male and female) endorsed tobacco use. The one-, three- and five-year cumulative mortality after the first hospitalization was 43.4%, 53.2%, and 61.6%, respectively for males and 24.1%, 59.0%, and 67.2%, respectively for females. Median survival for younger male patients with ALC (age < 40 years old) after the first hospitalization was significantly different compared to the older male patients (age > 40 years) (p=0.0009), but age was not a significant factor for survival of female patients.

Multivariate analysis further shows that illicit drug use, creatinine level at the time of admission, and age > 40 years had the highest hazard ratios for risk of mortality in male patients.

For female patients, history of hepatic encephalopathy (HE) and blood urea nitrogen (BUN) level at the time of discharge were both associated with increased risk of mortality, with a history of HE being associated with a higher hazard ratio for risk of mortality.

Conclusion

Age, illicit drug use, and creatinine level were risk factors associated with mortality for male patients with ALC but not female patients. Hepatic encephalopathy and BUN were risk factors associated with mortality for female patients. The mortality for male patients was about twice the mortality of female patients at one year, but three-year and five-year mortality was higher in female patients.

## Introduction

Alcohol use disorder (AUD) remains a significant health and socioeconomic burden on healthcare systems [1]. The World Health Organization (WHO) estimates 6% of all deaths worldwide to be related to alcohol consumption, although this data is based on death certificates and it may be an underrepresentation of globally distributed disease [2]. The harmful effects of alcohol are evident in multiple organ systems, including cardiovascular and gastrointestinal conditions, malignancies, and neuropsychiatric disorders [3-5]. The burden of alcohol use is the highest among liver diseases. Alcoholic liver cirrhosis (ALC) contributes up to 41% of alcohol-related liver deaths worldwide [2] and is the twelfth leading cause of death in the United States [6].

Unfortunately, mortality from ALC has been steadily increasing in multiple regions of the world including the U.S [7], and Europe [8]. Mortality from ALC has significantly increased with complications of portal hypertension and progression into decompensation: the presence of ascites, variceal hemorrhage, and hepatic encephalopathy (HE) [9]. One-year mortality in patients with compensated cirrhosis is 7% compared to 20% with decompensated cirrhosis [10].

Ascites is the most common complication of decompensated cirrhosis, found in up to 60% of previously compensated cirrhosis patients within the first 10 years [11]. The presence of ascites is also associated with increased mortality of 50% within the first three years [12]. Gastroesophageal varices are another manifestation of decompensated cirrhosis with the formation of collateral vessels that have an increased risk of rupture. Approximately 25-40% of gastrointestinal hemorrhages in cirrhotic patients are attributed to variceal bleed and each occurrence has a 10-30% rate of mortality [13,14]. The presence of overt HE is another evidence of cirrhosis decompensation with increased mortality rates. Overt HE has a prevalence of 16-21% in decompensated cirrhotic patients and increased in patients with a trans-jugular intrahepatic portosystemic shunt (TIPS) to 10-50% [15]. Over a five-year analysis, mortality due to HE in hospitals is about 15% [16].

Although the data on mortality and the associated risk factors of alcohol consumption continues to be topics of interest in the general population, there is limited literature on mortality based on patient’s sex. Traditionally, men suffer a higher number of alcohol-related deaths, but interestingly the rate of mortality has been increasing more in women than in men in recent years [17]. A study from 2009 to 2015 demonstrates a 50% increase in the incidence of alcohol-related liver injury in women, compared to a 30% increase among men during the same period [18]. Another meta-analysis further illustrates that despite similar quantities of alcohol consumption, cirrhosis is found more frequently in women versus men, attributing to increased mortality from alcohol consumption in females compared to males [19].

This study further compares the different risk factors for mortality, following diagnosis of alcoholic cirrhosis, between male and female patients. Earlier identification of these risk factors for each sex may facilitate targeted patient care based on severity, delay further progression of ALC, and improve hospital morbidity and mortality.

## Materials and methods

Study population

This study was approved by an institutional review board. It is a retrospective cohort study of patients with alcoholic cirrhosis who were treated during their first hospital admission for liver disease at Carilion Roanoke Memorial Hospital (CRMH), Roanoke, Virginia, USA; between August 1, 2008, and November 30, 2016, with follow-up through June 30, 2018. We included all identified patients ≥ 18 years of age diagnosed with alcoholic liver cirrhosis and collected data regarding demographics (age and sex), illicit drug use, lab values (hemoglobin and platelet counts, liver function tests, neutrophil-lymphocyte ratio [NLR], initial MELD [model for end-stage liver disease] score), and history of complications of end-stage liver disease (ESLD) including portal hypertensive hemorrhage, ascites, spontaneous bacterial peritonitis (SBP) and hepatic encephalopathy (HE). Initial admission data was used as the beginning date of the study. Late survival was defined as survival post-discharge up to five years.

Statistical analysis

The data were reported as mean (± standard deviation), median (interquartile range, IQR), ranges, and categorical variables by counts and percentages as appropriate. Estimates of the mortality rates were determined by using the Kaplan-Meier survival curve with a log-rank test. To identify risk factors associated with early mortality for each sex, we performed a univariate time-to-event analysis with Cox proportional regression models that accounted for the case-cohort design by using case weights to account for the sampling frame and robust estimates of variance. Variables with p < 0.05 on univariate analysis were included in a multivariate Cox proportional hazard analysis used to identify independent risk factors associated with mortality. All statistical analyses were conducted using JMP version 10 for Windows (SAS Institute Inc., Cary, North Carolina, United States).

## Results

Patient demographics

247 male patients and 78 female patients with alcoholic cirrhosis were identified. The mean age at the time of admission was 54.19 ± 13.14 years for male patients and 51.10 ± 11.60 years for female patients. Active alcohol use before admission was noted in 157 male patients (70%) and 33 female patients (46%); active illicit drug use was reported in 20 male patients (10%) and nine female patients (13%); active tobacco use was reported in 133 male patients (56%) and 41 female patients (56%).

Risk factors associated with mortality

For male patients, age >40 (Likelihood Ratio 11.11, p<0.05), illicit drug use (LR 6.26, p<0.05), blood urea nitrogen (BUN) level on admission (likelihood ratio [LR] 6.58, p<0.05), creatinine level on admission (LR 11.68, p<0.05), INR level on admission (LR 6.26, p<0.05), albumin level on admission (LR 7.42, p<0.05), WBC count on admission (LR 5.11, p<0.05), absolute neutrophil count on admission (LR 6.10, p<0.05), INR level on discharge (LR 6.82, p<0.05), lymphocyte count on discharge (LR 4.10, p<0.05) were all significant factors associated with increased mortality by univariate analysis. In a multivariate model including the stated risk factors from univariate analysis, age >40 (HR 2.58 [95% CI, 1.44-4.60], p=0.0014), illicit drug use (HR 118.65 [95% CI, 2.42-5806.52], p=0.0161), creatinine level on admission (HR 3.47 [95% CI, 1.64-8.42], p=0.0006), WBC count on admission (HR 1.45 [95% CI, 1.04-2.21], p=0.0238), and INR level on discharge (HR 2.47 [95% CI, 1.27-5.01], p=0.0090) were all associated with increased risk of mortality, with illicit drug use, creatinine level on admission and age >40 having the highest hazard ratio for increased risk of mortality (Table 1).

**Table 1:** risk factors for mortality among male patients of alcoholic cirrhosis BUN: blood urea nitrogen INR: international normalized ratio

Male Patients	Risk Factors of Mortality	Likelihood Ratio	Hazard Ratio [95% Confidence Interval]	P-value (p<0.05)
On Admission	Age > 40	11.11	2.58 [1.44-4.60]	0.0014
	Illicit Drug Use	6.26	118.65 [2.42-5806.52]	0.0161
	Initial BUN	6.58	0.941 [0.892-0.987]	0.0103
	Initial Creatinine	11.68	3.470 [1.642-8.423]	0.0006
	Initial INR	6.26	0.352 [0.142-0.802]	0.0124
	Initial Sodium	2.73	0.888 [0.759-1.021]	0.0988
	Initial Total Bilirubin	0.12	0.976 [0.849-1.116]	0.7285
	Initial Albumin	7.42	0.263 [0.094-0.689]	0.0065
	Initial White Blood Cell Count	5.11	1.445 [1.044 -2.211]	0.0238
	Initial Absolute Neutrophil Count	6.10	0.642 [0.399-0.922]	0.0135
	Initial Hemoglobin	1.36	1.210 [0.880-1.693]	0.2435
At Discharge	Last BUN	3.05	1.022 [0.997-1.049]	0.0808
	Last Creatinine	0.95	0.807 [0.516-1.240]	0.3298
	Last INR	6.82	2.467 [1.269-5.009]	0.009
	Last Total Bilirubin	0.19	0.971 [0.852-1.110]	0.6616
	Last White Blood Cell Count	0.99	0.937 [0.814-1.063]	0.3190
	Last Lymphocyte Count	4.10	0.899 [0.797-0.997]	0.0429
	Last Hemoglobin	1.10	0.802 [0.521-1.213]	0.2943

For female patients, a history of hepatic encephalopathy (LR 10.10, p<0.05) and BUN level at discharge (LR 4.86, p<0.05) were significant factors associated with increased mortality by univariate analysis. In a multivariate model including the stated risk factors from univariate analysis, history of hepatic encephalopathy (HR 6.54 [95% CI 1.82-23.52], p=0.0015) had the higher hazard ratio for increased risk of mortality compared to BUN level at discharge (HR 1.03 [95% CI, 1.00-1.06], p=0.0275) (Table 2).

**Table 2:** risk factors for mortality among female patients of alcoholic cirrhosis BUN: blood urea nitrogen

Female Patients	Risk Factors of Mortality	Likelihood Ratio	Hazard Ratio [95% Confidence Interval]	P-value (p<0.05)
On Admission	Age > 40	3.24	1.035 [0.997-1.077]	0.0717
	Hepatic Encephalopathy	10.10	6.542 [1.819-23.523]	0.0015
	Initial Total Bilirubin	0.01	1.004 [0.928-1.079]	0.9227
	Initial Albumin	0.90	0.758 [0.421-1.342]	0.3434
	Initial Hemoglobin	0.00	0.999 [0.827-1.199]	0.9944
At Discharge	Last BUN	4.86	1.034 [1.004-1.063]	0.0275
	Last White Blood Cell Count	0.65	0.868 [0.618-1.232]	0.4205
	Last Lymphocyte Count	1.34	0.956 [0.879-1.030]	0.2477
	Last Hemoglobin	3.59	0.764 [0.575-1.009]	0.0582

Comparison of long-term survival between male and female patients

We reviewed data over five years. For male patients, the mean follow-up after the first hospitalization was 2.96 ± 0.22 years (median 2.37 years [95% CI, 0.95-4.07]). The one, three, and five-year cumulative mortalities after the first hospitalization were 43.4%, 53.2%, and 61.6%, respectively for males.

For female patients, the mean follow-up after the first hospitalization was 2.39 ± 0.30 years (median 1.63 years [95% CI, 0.14-4.24]). The one, three, and five-year cumulative mortalities after the first hospitalization were 24.1%, 59.0%, and 67.2%, respectively for females.

**Figure 1:**
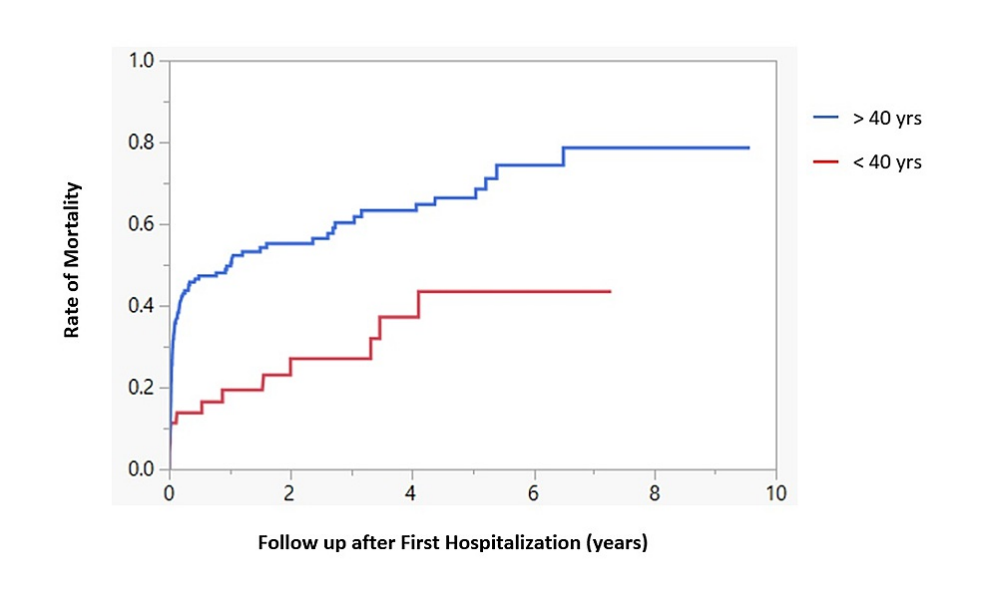
mortality among young vs. old male patients of ALC following first hospitalization

**Figure 2:**
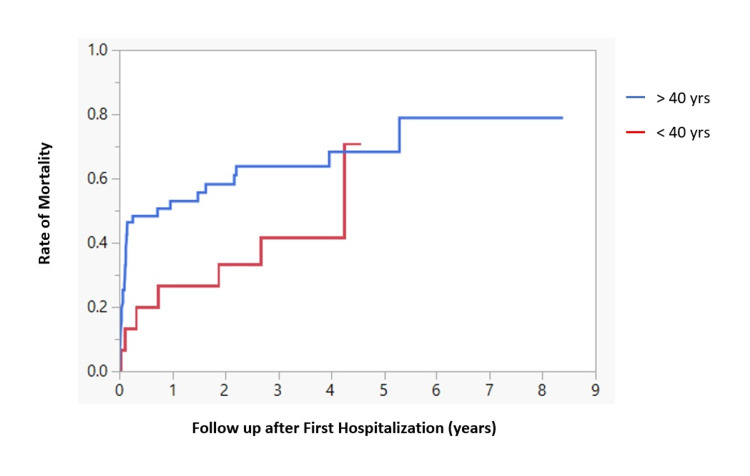
mortality among young vs. old female patients of ALC following first hospitalization

Median survival for younger male patients with ALC (age <40 years old) after the first hospitalization was statistically significant compared to the older male patients (age >40 years) (p=0.0009) (Figure 1), but age was not a significant factor for survival of female patients (p=0.133) (Figure 2).

## Discussion

In this study of a nearly decade-long sample of hospitalized patients in Southwest Virginia, USA with alcoholic cirrhosis, we found differences in risk factors comparing male and female patients that lead to increased risk of mortality. In males, age > 40, illicit drug use, creatinine level on admission, WBC count on admission, and INR level on discharge were all associated with increased risk of mortality, with illicit drug use, creatinine level on admission and age > 40 having the highest hazard ratio for increased risk of mortality. On the other hand, for females, a history of hepatic encephalopathy and BUN levels at discharge were associated with increased risk of mortality, with the history of hepatic encephalopathy having the higher hazard ratio.

INR and total bilirubin are other laboratory values commonly included in current prognostic scoring systems such as Child-Turcotte-Pugh and MELD. However, when examined separately among the two sexes, both INR and total bilirubin were not statistically significant risk factors of mortality in female patients. For male patients, they were both associated with increased risk of mortality, but their hazard ratios were not as high (HR 2.47 and 0.98 respectively for INR and total bilirubin) as other risk factors including illicit drug use, creatinine level on admission, and age > 40. One may speculate that the current prognostic scoring systems may reflect risk factors associated with mortality in male patients more than that of female patients given their higher prevalence of alcoholic cirrhosis. Interestingly, this study reflects that different risk factors may be associated with the risk of mortality when the patient population is examined more specifically based on sex.

It is not surprising that male and female patients have different risk factors associated with increased mortality in ALC as women are more susceptible to alcohol-related liver injury [20]. In a meta-analysis of 2.5 million participants Roerecke et al. report that the consumption of even one drink per day compared to long-term abstainers showed an increased risk of liver cirrhosis in women, but not in men [17]. It is not surprising then that the Dietary Guidelines for Americans 2015-2020 advise the two sexes to have different recommendations for “safe” levels of alcohol consumption: women should not consume more than 14 grams of alcohol daily, while men should not consume more than 28 grams of alcohol daily [21].

There are subtle differences between the sexes that put women at a higher risk of alcohol-related liver injury when compared to men. Women tend to have decreased body water content compared to men, leading to a higher concentration of blood alcohol level (BAL) with similar consumption of alcohol [22]. Further studies show differences in expression of hepatic enzymes between two sexes such as under-expression of cytochrome P450 2E1 as well as decreased gastric alcohol dehydrogenase in women, thus decelerating the degradation of blood alcohol, compared to men [23]. Female patients thus would have higher BAL despite similar consumption to males and therefore are at increased risk for alcohol-related multi-organ damage, including liver diseases and ALC.

Identification of gender-specific risk factors associated with ALC is crucial for a personalized assessment of the severity of the alcohol-related liver injury and if appropriate, early referral for a liver-transplant evaluation. Unfortunately, the prevalence of alcohol-related liver injury including ALC has been increasing. Consequently, the demand for liver transplants has been increasingly difficult to accommodate, leading to a longer waiting period. Complications from portal hypertension and subsequent hospital admission are common among patients with cirrhosis [24]. Hospitalization in patients with cirrhosis is also associated with increased mortality. Interestingly, a 12-month study completed by Rubin et al found that female patients with cirrhosis on the liver transplant waitlist tend to have a higher risk of hospitalization compared to males (OR 1.6 [95% CI, 1.1-2.6], p=0.03). In addition, female patients had higher median number of total inpatient days compared to males (OR 2.5 days [95% CI: 0-10.0] vs. OR 0 days [95% CI: 0-6.5]; p=0.02) [25]. Furthermore, a review of data from U.S SRTR (Scientific Registry of Transplant Recipients) by Sarkar et al also illustrates that female patients had higher risks of mortality while on the waitlist for liver transplant than the male patients (HR 1.3; [95% CI: 1.1-1.5]; p=0.003) [26].

A plausible explanation for the different outcomes of patients on the liver transplant waitlist based on sex is that the female patients had a higher rate of mortality at the time of transplant enlistment or developed more rapid progression of cirrhosis during the waiting period. However, the study suggests that women have similar or even lower MELD scores at listing compared with men, suggesting they did not have higher estimated mortality rates at baseline [25]. In a study of patients registered on the UNOS ( United Network for Organ Sharing) liver transplantation waiting list pre- and post-MELD adaptation by Moylan et al, female patients continued to experience approximately 30% increased odds of death or becoming too sick for liver transplantation compared to males even after adjusting for MELD score at the time of listing [27]. Then, female patients may have an increased risk of rapid progression of cirrhosis given that female patients were at greater risk of hospitalization and more inpatient days.

Our study of patients with ALC who are not on the transplant waiting list similarly validates that female patients with a history of ALC have an increased rate of mortality compared to male patients with longer follow-up. After one year of follow-up, the mortality rate of female patients with ALC after the first hospitalization was 24.1% compared to 43.4% in male patients. However, there was a steep incline in the rate of mortality in female patients at three-year follow-up, with a mortality rate of 59.0% (from 24.1% at one year) compared to only a modest increase in mortality of male patients to 53.2% (from 43.4% at one year). Even at five-year follow-up, the mortality rate of female patients continued to rise above that of the male patients (67.2% vs. 61.6%).

One of the leading hypotheses for higher risk of mortality of female cirrhotic patients compared to males is the underestimation of renal function in women due to their relative decreased muscle mass when compared to men. This results in lower creatinine levels for females with similar degrees of renal impairment compared to males, leading to lower MELD scores [28]. Although the level of creatinine was not a risk factor for increased mortality in our female patients with alcoholic cirrhosis, the level of BUN at discharge was associated with increased risk of mortality in females (HR 1.03, p=0.0275), indicating that the impact of renal dysfunction may be different between the two sexes and benefit from further evaluation. Another proposal is that the female patients remain on the transplant waitlist for longer despite similar severity due to their relatively smaller physical stature compared to men. Many transplant organs are harvested from deceased donors and statistically, more likely to be from deceased male patients than females. After donor-recipient size-matching, females are less likely to receive the donated organs than males based on their stature. Even when smaller organs are available, the limited pool is shared between female and child recipients [29]. Still, neither hypotheses provide a comprehensive explanation for the more rapid progression of cirrhosis in females compared to males and a more targeted study design in the future may be beneficial.

The prevalence of alcohol-related liver injuries and cirrhosis continues to rise, where liver transplant still is the optimal treatment for end-stage liver disease. Yet sex disparities in access to transplant continues to persist. Current scoring systems for understanding mortality and transplant eligibility in patients with ALC do not consider sex-specific differences. Neither the MELD score used for grading the severity of end-stage liver disease and determining transplant eligibility nor the Child-Turcotte-Pugh score identifying cirrhosis severity identify sex as a risk for poor prognosis [27]. Identifying the sex-specific risk factors of mortality is important for predicting which patients are at increased risk of mortality. With an improved risk-stratification model, patients with a greater risk of mortality can be prioritized for closer monitoring by healthcare professionals and earlier liver transplant evaluation.

The retrospective nature of the collected data, the small sample size with an uneven distribution of the two sexes, and the localization to a single-center are limitations to this study. We also were not able to collect information on patients who were admitted previously to other facilities due to logistical reasons. In addition, demographic information on race was also limited within our EMR. Our results therefore may not be representative of nationwide demographics in terms of quantity of alcohol consumption, duration of alcohol abstinence, and drinking status after discharge. These factors could significantly affect the conclusions regarding mortality after discharge. Another limitation of this study is that we focused on patients undergoing hospital admission and did not include patients with ALC who followed up in the clinic, and did not require hospitalization for decompensation. Further retrospective and prospective studies are needed to identify additional sex-specific risk factors impacting mortality in patients with ALC. We also were not able to quantify alcohol use which may present as a limiting factor as gender-specific differences in alcohol consumption do impact the development of chronic liver disease.

## Conclusions

In conclusion, this study illustrates several different risk factors associated with unfavorable outcomes when comparing male and female patients with ALC. Age, illicit drug use, and creatinine level were risk factors for male but not female patients. Hepatic encephalopathy and BUN were risk factors for female patients. Furthermore, both sexes showed a significant rate of mortality at one, three, and five years. However, female patients illustrated a dauntingly rapid rate of decline when comparing one-year mortality to three- and five-year mortalities and also when comparing to mortalities of males. Patients of either sex with these identified risk factors, especially for females, should be monitored aggressively and referred early for liver transplant evaluation.
